# Anticipatory Postural Adjustments in Dart Throwing

**DOI:** 10.2478/hukin-2013-0023

**Published:** 2013-07-05

**Authors:** Grzegorz Juras, Kajetan Słomka

**Affiliations:** 1 Department of Motor Behavior, The Jerzy Kukuczka Academy of Physical Education in Katowice, Poland.

**Keywords:** speed-accuracy trade-off, Fitts’ law, posture control, anticipatory postural adjustment

## Abstract

The aim of this study was to explore the effects of accuracy constraints on the characteristics of anticipatory postural adjustments (APA) in a task that involves a movement consisting of a controlled phase and a ballistic phase. It was hypothesized that APA scaling with task parameters (target size) would be preserved even when the task is performed by muscles that have no direct effects on APA. Sixteen healthy right handed subjects participated in the study. All participants had no prior experience in dart throwing. Subjects’ average age was 24.1 ± 1.9 years. A force platform and a motion capture system were used to register kinetics of the body and kinematics of the throwing arm and throwing accuracy. The experiment consisted of six series of twenty consecutive dart throws to a specified target. Target sizes (T2–T6) were set at 25%, 50%, 75%, 125% and 150% of target 1 (T1) initially set as the spread of the last 20 throws in a 50 throw training session. This allowed to distinguish six indexes of difficulty (ID’s) ranging from 2,9 to 5,9. A one-way ANOVA for repeated measures was used for statistical analysis. Results of ANOVA showed a significant effect of target size at Constant Error but no effect at APA time. There were also no significant differences between hit and miss throws. From a control perspective, it can be stated that changes in central commands did not lead to changes in APA time in the analyzed motor task.

## Introduction

There is a specific relation between movement speed and accuracy which can be observed in many situations of our life. Every time we try to be more accurate we have to slow down. It works in the opposite direction as well – when we want to speed up, we have to compromise the accuracy of our movements. This phenomenon is called speed-accuracy trade-off and is described by Fitts’ law ( Fitts, 1954 ) which is one of the most respected quantitative laws of motor behavior. The law links movement time (MT) to movement distance (D) and target size (W). Commonly, Fitts’ law is written as: MT = a + b • log2 (2D/W), where a and b are constants. The value log2 (2D/W) is called the index of difficulty.

Both from the theoretical and empirical point of view Fitts’ law successfully describes the speed-accuracy trade-off in cyclic tasks. However, that phenomenon in single rapid movements is still far from understood ( Plamondon and Alimi, 1997 ).

When a standing person gets ready to perform a quick arm action, changes in the activation of leg and trunk muscles are seen prior to movement initiation ( Aruin and Latash, 1995 ; Belenkiy et al., 1967 ; Cordo and Nashner, 1982 ). Such changes have been addressed as anticipatory postural adjustments (APAs, reviewed in Massion, 1992 ). The purpose of APAs has been assumed to generate forces and moments of force acting against those expected from the planned movement due to the mechanical coupling of the body segments. Several recent studies have shown that APAs scale with task accuracy requirements when a person is performing the movements “as fast and accurately as possible” ( Bertucco et al, 2013 ; Bertucco and Cesari, 2010 ; Duarte and Latash, 2007 ). These observations suggest, in particular, that the famous speed-accuracy trade-off ( Fitts, 1954 ) is not due to corrections of ongoing movements ( Meyer et al., 1990 ) but emerges at the level of movement planning ( Latash and Gutman, 1993 ).

The purpose of the current study was to explore the effects of accuracy constraints on the characteristics of APAs in a task that involves a movement consisting of a controlled phase and a ballistic phase. We selected the dart throwing task because of the clear separation of the two phases. Note that accuracy of hitting the target during dart throwing is defined by selecting proper combinations of the magnitude and angle of the release velocity vector, and the point of dart release. As such, this task belongs to the class of redundant tasks ( Bernstein, 1967 ) because an infinite number of combinations of the mentioned parameters can result in the same dart coordinate when it crosses the plane of target.

Changes in target size were expected to lead to scaling of the velocity profile during the controlled phase of the throw. Based on the mentioned studies that showed APA scaling with action velocity, it was hypothesized that the APAs would scale with target size.

## Material and Methods

### Participants

The study was conducted on sixteen healthy male (10) and female (6) right handed Physical Education students. Their average age was 24.1 ± 1.9 years [mean ± SD], body height and mass were respectively: 1.73 ± 0.059 m and 70.2 ± 8.6 kg. All participants had no prior experience in dart throwing. They had no neurological, musculoskeletal or any other postural disorders. The experiment was approved by the Institutional Bioethics Committee. Subjects agreed to participate in the experiment voluntarily. The purpose of the study was explained to them and written informed consent was obtained from all participants.

### Apparatus

A force platform (AMTI, BP600900) was used in the experiment to measure the vertical component of ground reaction forces (Fz). The signal was sampled at 1 kHz. At the same time kinematics of a throwing arm and hit accuracy were registered using passive markers with infrared cameras (BTS Bioengineering, BTS Smart). Markers were placed at arm, elbow and wrist joints and at the end of a dart. Target was positioned 2 m away from the subject. The width of the target was constant and equaled 1m, while its height varied across the series.

### Procedure

The experiment consisted of six series of twenty consecutive darts throws to specified target. Target sizes (T2–T6) were set at 25%, 50%, 75%, 125% and 150% of target 1 (T1) initially set as the spread of the last 20 throws in a 50 throw training session. This allowed to distinguish six indexes of difficulty (ID’s) ranging from 2,9 to 5,9. The following variables were used in further analysis: anticipatory postural adjustment time (tAPA - the time that elapses between movement initiation (when the magnitude of Fz deviated from Q by more than 10% in either direction) and arm movement initiation), time of movement (tMOV), flight time of a dart (tFlight), overall time (tALL), distance between average dart location and the centre of the target (Constant Error) and the standard deviation of the target coordinates across trials (Variable Error).

**Figure 1:**
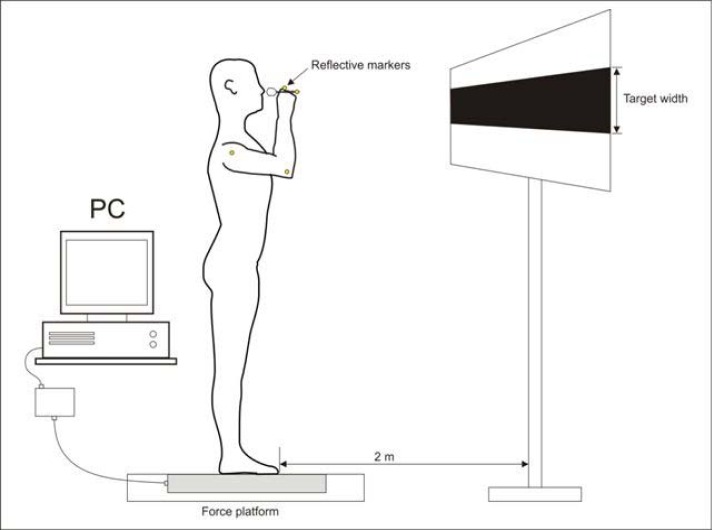
*
Experimental setup
*

The subjects were instructed to throw in a self-paced manner, any time within 10 s after a “ready” signal. In the initial position, the subject was standing prone and comfortable with the dominant hand handling a dart closest to the ear ( Figure 1 ). A procedure was randomized. After each series there was a 2 min break to avoid fatigue and to mark a new width of aim. During this time the subjects were asked to rest in a standing position. Subjects did not report fatigue during the whole experiment.

### Data processing

MATLAB (The Mathworks, Natick, MA) software package was used to process the data. The vertical component of ground reaction forces (Fz) was used to estimate the subject’s weight (Q) by averaging data over 0.5 s while the subject stood quietly. Movement initiation time (TSTART) was defined as the time when FZ deviated from Q by more than 10%. The take-off time (TOFF) was defined as the time when FZ became zero. The landing time (TLAND) was defined as the time when the first contact was made by one of the electrodes and the contact plate. Movement time was defined as: MT = TLAND – TOFF. Anticipatory postural adjustment time was defined as: TAPA = TOFF – TSTART.

### Statistics

Standard methods of descriptive statistics were applied in the present study. The Shapiro-Wilk and Lilliefors tests were used to check the data for normal distribution, while variance homogeneity was investigated with the Levene’s test. One-way ANOVAs with repeated measures was used to analyse MT and TAPA with the factor Width (six levels). All data were expressed as mean ± SD. The significance level was set at p<0.05.

## Results

**Figure 2:**
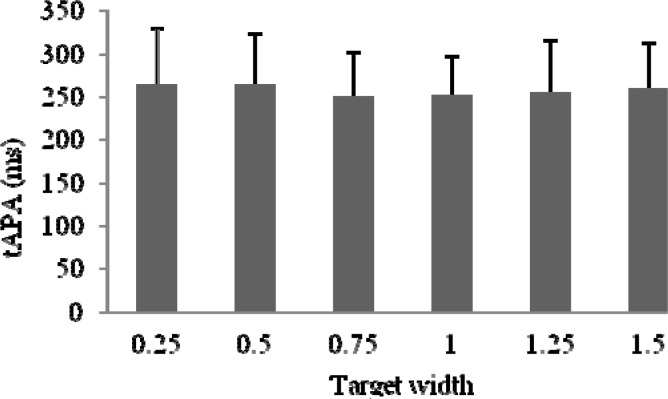
*
Mean tAPA across 20 throws independently of its accuracy for all subjects
*

**Figure 3:**
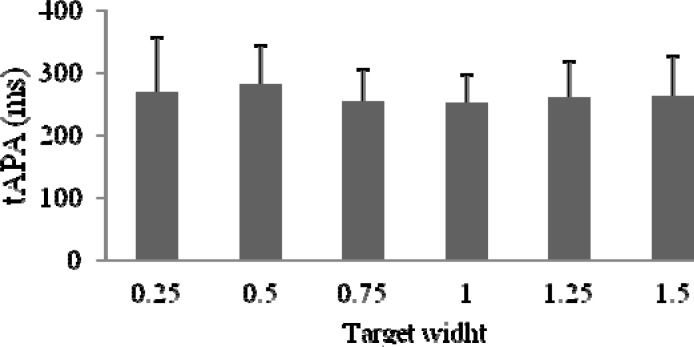
*
Mean tAPA for hit throws only
*

First, a one-way ANOVA was conducted to compare the effect of target size on the anticipatory adjustment time (tAPA). There was no significant effect of target size both for all throws (F (1,14) = 0,5707) and hit ones only (F(1,14) = 0,6192). The plotted data representing 20 throws (independent of the hit accuracy) and the hit targets throws only were presented in Figures 2 and 3 .

**Figure 4:**
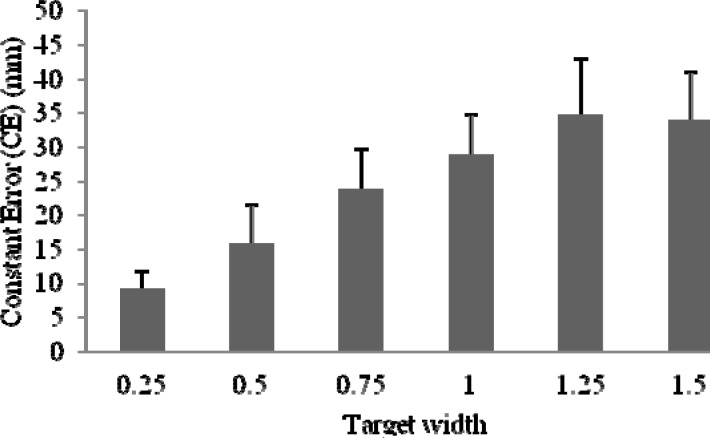
*
Mean Constant Error for hit throws only
*

No significant differences were observed also for variables describing the kinematics of the throwing task. Both differences between mean values of movement time (the time between the first registered movement of either of the two markers and the moment of dart release - tMOV) and mean values of flight time of a dart (tFlight) measured for different sizes of target were not significant. The only observed significant difference was a constant error (CE) for hit throws (F(1,14) = 2,7438; p<0,05). These data were presented in Figure 4 . It is possible to notice that the distance between average dart location and the center of the target had significantly smaller magnitudes for the more difficult task (the smallest target – the smallest constant error). It could be interpreted as a natural effect of target size and a conducted research procedure.

## Discussion

Over the past 50 years, Fitts’ law has been supported in a large number of experimental studies involving different tasks, effectors, force fields, and subject populations ( Plamondon and Alimi, 1997 ). Several recent studies ( Freitas et al., 2007 ; Duarte and Freitas, 2005 ; Duarte and Latash, 2007 ; Gorniak et al., 2008 ; Juras et al., 2009 ) have cast doubt on the universal nature of Fitts’ law by showing that the dependence of movement time (MT) on the width (W) and distance (D) could not be reduced to a single equation: MT depended on W, as predicted by Fitts’ law, but only for a given distance. When D is changed, the coefficients *a* and *b* in the equation are changed as well.

There are only few studies that examine activity of APA with Fitts’ law. To the authors knowledge, there is only one study presented by Bertucco and Cesari (2010) who provided detailed research on a typical dance movement ( *battement tendu* ). They showed that APA onset and magnitude are independently organized by the CNS through different pre-planning strategies. They noticed that the greater the distance, the greater the velocity and the APA magnitude. Simultaneously, within each distance the APA magnitude decreases as the demand for accuracy increases ( Bartucco and Cesari, 2010 ). No similar changes with target size were observed in the present study.

The aim of this study was to investigate the effect of target size on the anticipatory adjustment time in a throwing task. It was expected that the APAs would scale with the target size. The results of this study do not support our hypothesis. It was thought that the subjects would prolong the preparation phase when they faced smaller targets but they ultimately reached similar velocity vectors leading to similar MT values for throwing over the same distance but different target size. The ballistic nature of the task might have mitigated the scaling of APA time with target size because the subjects were aware that we were trying to affect MT. That possible explanation proves that it is hard to exclude psychological aspects from any human movement.

Another explanation is related to biomechanical laws and muscle properties. Subjects were asked to throw from the same distance (2 meters) and probably the same level of force recruitment together with peak force was observed. If so, elastic characteristics of muscles and tendons served as a self-control mechanism. That hypothesis should be confirmed in follow up studies focused on different discrete movements. Our results do not confirm data presented by Adam et al. (1993) who noticed that cyclical movements can have certain discrete characteristics if subjects meet high spatial-accuracy constraints. According to their findings Fitts’ law might be limited to egocentric visuomotor actions. Smyrnis et al. (2000) also showed that different movement parameters are affected by target distance, size and direction. Their results indicate that there is a crucial distinction between parameters affected by target size and direction. Furthermore, the number of secondary peaks on the speed record increased with decreasing target size ( Smyrnis et al., 2000 ).

From a control perspective, it can be stated that changes in central commands did not lead to changes in APA time in the analyzed motor task. Therefore, one should remember that it was a rapid movement which differs from cyclic ones. However, Winstein et al. (1997) found that in classical tapping tasks, when more precise targeting independent of task difficulty was required, a cortical-subcortical loop composed of the contralateral motor cortex, intraparietal sulcus and caudate was much more activated. They showed, with a use of positron emission tomography (PET), that greater effort in performing a difficult task (smaller targets) recruits more motor planning areas. Recent studies showed that there is a specific modulation of neural network associated with the availability of time to plan the upcoming movement and motor difficulty. One of them used brain-imaging (fMRI) to examine a simple motor task - moving a mouse cursor on a screen ( Boyd et al., 2009 ). Another examined step initiation in patients with Parkinson’s disease ( Jacobs et al., 2009 ). The same concerns the study by Bartucco and Cesari (2010) described earlier, which focused on motion capture experiments on ballet movements. It looks like in these experiments subjects used distinct control of APA duration and APA magnitude according to Fitts’ law. It is one of the limitation of our study that we did not observe changes in the central nervous system. An additional limitation is that we did not record muscle activity.

It is hard to estimate information processing but it can be guessed that the commands do not concern speed manifested in the velocity of a dart but the accuracy of aiming. Concentrating on accuracy does not have to lead to changes in force recruitment. That hypothesis is partly supported by Smits-Engelsman et al. (2002) who suggest fundamental differences in cyclic and discrete movements. They also claim that cyclic movements make a more cost-effective use of the recruited force, use less information-processing capacity and less change in force, then discrete ( Smits-Engelsman et al., 2002 ). This interesting hypothesis is worth considering and examining in future research.

Whenever we optimize the speed-accuracy trade-off in specific movement by repetitions we can create a motor skill and perform the movement better and better. Then we start to act effortless and automatic. Unfortunately, there is a lack of data concerning some applications of Fitts’ law in sports training. It is simply impossible to say if it is better to differentiate a distance or a target size during the process of gradual mastering of specific motor skills with repeated performance. From a physics point of view, controlling velocity seems to be the simplest way to perform a motor task. It may be more effective to change spatial constraints to achieve better results in high-performance sport. Although neuroscience has made major advances within the past 10–20 years, creating a perfect basketball or golf player is still a challenge.
